# Integrated multi-omics analysis reveals gut microbiota and metabolic characteristics in coronary heart disease

**DOI:** 10.3389/fmicb.2026.1743914

**Published:** 2026-03-10

**Authors:** Liqi Peng, Yuanting Zhang, Xudong Li, Zongren Hu

**Affiliations:** 1 Affiliated Hospital of Nanjing University of Chinese Medicine, Nanjing, Jiangsu, China; 2 The First Clinical Medical College of Nanjing University of Chinese Medicine, Nanjing, Jiangsu, China; 3 Postgraduate Medical Education Center, The First Affiliated Hospital of Hunan University of Chinese Medicine, Changsha, Hunan, China; 4 School of Integrated Chinese and Western Medicine, Hunan University of Chinese Medicine, Changsha, Hunan, China; 5 School of Traditional Chinese Medicine, Hunan University of Medicine, Huaihua, Hunan, China; 6 Hunan Engineering Research Center of Internet-Chinese and Western Medicine Collaboration-Health Service, Hunan University of Medicine, Huaihua, Hunan, China

**Keywords:** biomarkers, coronary heart disease, gut microbiota, metabolomics, multi-omics, proteomics

## Abstract

**Background:**

Coronary heart disease (CHD) is a leading cause of morbidity and mortality worldwide. Increasing evidence indicates that gut microbiota dysbiosis contributes to CHD pathogenesis through metabolic, inflammatory, and coagulation-related mechanisms. However, comprehensive multi-omics investigations of individuals with CHD remain limited. In this study, we aimed to characterize the multi-omics features of CHD and to identify potential diagnostic biomarkers.

**Methods:**

The study included 10 patients with clinically diagnosed CHD and 10 healthy controls. Blood and fecal samples were collected for further analysis. The gut microbiota composition was assessed using 16S ribosomal RNA high-throughput sequencing, and shotgun metagenomic sequencing was further performed to evaluate microbial functional potential through the Kyoto Encyclopedia of Genes and Genomes (KEGG) annotation and differential pathway analysis. Non-targeted metabolomic profiling was performed using ultra-high-performance liquid chromatography coupled with Orbitrap mass spectrometry, and quantitative proteomic analysis was conducted using liquid chromatography–tandem mass spectrometry. Functional interaction networks between differentially expressed metabolites and proteins were constructed using Spearman correlation analysis, and the diagnostic potential of candidate biomarkers was evaluated using receiver operating characteristic (ROC) curve analysis.

**Results:**

At the phylum level, the CHD group exhibited an increased abundance of *Pseudomonadota* and a decreased abundance of *Bacillota* and *Actinomycetota*. At the genus level, *Escherichia–Shigella, Bacteroides*, and *Klebsiella* were significantly enriched, whereas *Bifidobacterium* and *Faecalibacterium* were decreased in abundance. Shotgun metagenomic analysis revealed functional remodeling of gut microbiota in CHD, with upregulation of KEGG pathways related to energy metabolism, inflammatory signaling, and host–microbe interactions. Serum metabolomics and proteomic analyses identified 32 differentially expressed metabolites and 38 differentially expressed proteins, respectively. Correlation analysis revealed significant associations between phospholipid metabolites and apolipoproteins, inflammatory mediators and the complement system, asymmetric dimethylarginine and endothelial function–related proteins, and oxidative stress metabolites and antioxidant proteins. ROC analysis identified several potential biomarkers with high diagnostic value.

**Conclusion:**

We demonstrate that individuals with CHD exhibit significant gut microbiota dysbiosis, distinct metabolic pathway alterations, and aberrant expression of coagulation- and inflammatory-related proteins. These findings provide novel insights into potential targets for CHD prevention and treatment strategies.

## 1 Introduction

Coronary heart disease (CHD) is a major cause of mortality and disability worldwide. According to the latest statistics from the World Health Organization, approximately 18.6 million people die from cardiovascular diseases each year, with CHD accounting for nearly 45% of these deaths (World Health Organization, 2023). The 2024 China Cardiovascular Health and Disease Report Summary indicated that the number of patients with CHD in China exceeded 8.92 million (National center for cardiovascular diseases the writing committee of the report on cardiovascular health diseases in China, 2024), showing a continuous upward trend in incidence. In recent years, clinical interventions such as statins for lipid regulation, antiplatelet therapy, and revascularization techniques have significantly improved CHD management by slowing disease progression and enhancing patient outcomes (Wu et al., 2024; Tantry et al., 2026; Cao et al., 2025). However, these therapeutic strategies still face challenges in accurately detecting CHD in its early stages, enabling individualized treatment, and reducing the readmission rates. To further improve CHD prevention and management, identifying molecular biomarkers and therapeutic targets that can facilitate early diagnosis and precision interventions through an in-depth understanding of the underlying molecular mechanisms of the disease is essential.

Atherosclerosis constitutes the fundamental pathological basis of CHD, with its initiation and progression driven by multiple interrelated mechanisms, including lipid metabolism disorders, chronic inflammation, oxidative stress, platelet activation, and endothelial dysfunction (Libby, 2021; Gimbrone and García-Cardeña, 2016). Low-density lipoprotein cholesterol (LDL-C) accumulates in the arterial intima and undergoes oxidation, triggering macrophage phagocytosis and foam cell formation, which further promotes the release of inflammatory mediators and the development of atherosclerotic plaques (Tabas, 2010). As plaques become unstable, they may rupture and induce acute coronary events (Virmani et al., 2006). Recent evidence has highlighted the critical role of the gut microbiota and its metabolites in the pathogenesis and progression of CHD (Abdulrahim et al., 2025; Escobar et al., 2025). Trimethylamine (TMA), generated by gut microbiota metabolism of dietary choline and carnitine, is oxidized in the liver to form trimethylamine N-oxide (TMAO). TMAO promotes atherosclerosis progression, enhances platelet reactivity, and increases the risk of adverse cardiovascular events (Wang et al., 2011; Koeth et al., 2013). Therefore, alterations in the composition and function of the gut microbiota may provide critical biological information for early detection and stratification of CHD. The rapid advancement of multi-omics technologies in recent years has provided powerful tools for elucidating the complex molecular mechanisms underlying CHD. Metabolomics enables comprehensive profiling of small-molecule metabolites in plasma, urine, or feces, offering insights into systemic metabolic states and their dynamic alterations (Nordestgaard et al., 2025). Meanwhile, proteomics enables global analysis of protein expression, post-translational modifications, and associated signaling pathways, facilitating the identification of key pathogenic proteins and potential therapeutic targets (Lam et al., 2016). Despite growing evidence implicating the gut microbiota, metabolomic, and proteomic changes in CHD, most existing studies remain confined to a single-omics level (Chen et al., 2023a; Vilne and Schunkert, 2018). This limitation makes it challenging to systematically elucidate the interconnections among different biological levels, as single-omics approaches often capture only partial aspects of a disease. CHD is a multifaceted condition influenced by a wide range of factors and pathways. Its onset and progression result from a complex interplay of mechanisms, including imbalances in gut microbiota, metabolic disturbances involving small molecules, and aberrant protein signaling networks (Tang et al., 2017). Consequently, integrating gut microbiota, metabolomic, and proteomic data enables a multidimensional characterization of the molecular landscape of CHD, allowing the identification of biomarker combinations that reflect cross-system synergistic interactions. This integrative approach holds great potential for improving the accuracy of early disease diagnosis and risk prediction.

Accordingly, we aimed to investigate the multi-level molecular characteristics of patients with CHD by integrating gut microbiota, metabolomic, and proteomic data. Specifically, we sought to characterize the gut microbial composition and metabolic alterations in CHD, identify differentially expressed proteins (DEPs) and their associated metabolic pathways, and explore potential cross-talk across multiple biological layers. Through multi-omics integration, we seek to provide comprehensive insights into the molecular mechanisms underlying CHD, thereby contributing to early disease identification and the discovery of novel biomarkers and therapeutic targets.

## 2 Materials and methods

### 2.1 Study population

The present study was approved by the Ethics Committee of the First Affiliated Hospital of Hunan University of Chinese Medicine (approval No: HN-LL-YJSLY-2024-067), and all participants provided written informed consent. Following this approval, 10 patients newly diagnosed with CHD were recruited from the Department of Cardiology (CHD group). All participants met the diagnostic criteria for CHD established by the American Heart Association and the American College of Cardiology (Virani et al., 2023). All patients were newly diagnosed and had not received any pharmacological treatment prior to their enrollment. Additionally, 10 healthy individuals were recruited as the normal control (NC) group. Healthy controls were recruited from individuals undergoing routine health examinations at the Health Management Center of the same hospital, and were confirmed to be healthy based on physical examination reports and medical records, with no history of CHD or other major chronic systemic diseases. Participants were excluded if they met any of the following criteria: use of antibiotics, probiotics, or prebiotics within the past 3 months; severe diarrhea; concomitant diabetes, kidney disease, other systemic disorders, or psychiatric disorders; or pregnancy. Lifestyle factors such as smoking status, alcohol consumption, and detailed dietary patterns were not systematically recorded, and are acknowledged as potential confounding factors.

### 2.2 Sample collection and processing

All samples were collected in the early morning after the participants fasted for at least 8 h. Female participants avoided sample collection during their menstruation. Five mL of venous blood was drawn into vacuum tubes and allowed to stand at room temperature for 2 h. Samples were then centrifuged at 3,000 rpm for 15 min at 4 °C. The supernatant was transferred into 1.5 mL cryogenic tubes and stored at −80 °C until further analysis. Fecal samples were collected using sterile swabs, with approximately 3 g obtained from each participant, ensuring the absence of urine and other contaminants. Following collection, fecal samples were promptly transferred to 5 mL centrifuge tubes, rapidly frozen in liquid nitrogen, and stored at −80 °C to prevent repeated freeze–thaw cycles that could compromise sample quality.

### 2.3 Biochemical indicators

Serum biochemical parameters were measured in all participants using a fully automated biochemical analyzer (Chemray 800; Rayto Life and Analytical Sciences Co., Ltd., Shenzhen, China). The measured parameters included alanine aminotransferase (ALT), aspartate aminotransferase (AST), alkaline phosphatase (ALP), γ-glutamyltransferase (GGT), albumin (ALB), total bilirubin (TBIL), direct bilirubin (DBIL), indirect bilirubin (IBIL), glucose (GLU), urea (UREA), creatinine (CREA), uric acid (UA), blood urea nitrogen (BUN), creatine kinase (CK), creatine kinase isoenzyme MB (CK-MB), lactate dehydrogenase (LDH), triglycerides (TG), cholesterol (CHO), high-density lipoprotein cholesterol (HDL-C), and LDL-C.

### 2.4 Metagenomic DNA extraction and quality control

Total genomic DNA was extracted from fecal samples using the OMEGA Soil DNA Kit (D5635**–**02) (Omega Bio-Tek, Norcross, GA, USA), with all procedures strictly adhering to the kit instructions. The extracted DNA was used for shotgun metagenomic sequencing. DNA integrity and concentration were assessed using the Agilent 5,400 system (Agilent Technologies, USA) to ensure suitability for library preparation and sequencing.

### 2.5 16S ribosomal RNA gene sequencing and analysis

For 16S ribosomal RNA (rRNA) gene sequencing, total DNA was extracted from the same fecal samples using the cetyltrimethylammonium bromide method. DNA quality was assessed using agarose gel electrophoresis, and the concentration was quantified using a UV spectrophotometer. The V4 region of the bacterial 16S rRNA gene was amplified by polymerase chain reaction (PCR) using the forward primer 515F [(5′-GTGYCAGCMGCCGCGGTAA3′)] and reverse primer 806R [(5′-GGACTACHVGGGTWTCTAAT3′)] (Walters et al., 2016). PCR amplification was performed under the following conditions: initial denaturation at 98 °C for 30 s; 32 cycles of denaturation at 98 °C for 10 s; annealing at 54 °C for 30 s; and extension at 72 °C for 45 s. PCR products were verified on a 2% agarose gel, purified using AMPure XT beads (Beckman Coulter Genomics, Danvers, MA, USA), and quantified using a Qubit fluorometer (Invitrogen, USA). The purified products were re-evaluated using 2% agarose gel electrophoresis before pooling for high-throughput sequencing. Sequencing data underwent adapter and barcode removal, sequence merging, quality filtering, and denoising using DADA2. Subsequently, alpha, and beta diversity analyses, taxonomic annotation, and differential abundance analyses were conducted, followed by downstream functional analyses to explore the community structure and functional differences.

### 2.6 Library preparation and metagenomic sequencing

A paired-end sequencing strategy was employed for metagenomic library construction. Genomic DNA was randomly sheared into fragments of approximately 350 bp average length using a Covaris ultrasonic homogenizer (Covaris, Woburn, MA, USA). Library preparation was completed through sequential steps including end repair, A-tailing, adapter ligation, purification, and PCR amplification. Based on effective library concentrations and target read yields, multiple libraries were pooled and subjected to high-throughput sequencing on the Illumina NovaSeq X Plus PE150 platform (Illumina, San Diego, CA, USA). Raw sequencing data underwent quality control (QC) using Fastq (v0.23.2) to remove adapter sequences and low-quality reads (Chen et al., 2018). Subsequently, high-quality reads were aligned against the human reference genome using Bowtie2 (v2.5.1) to eliminate host-derived sequences (Langmead and Salzberg, 2012). Host-contaminated sequences were removed, and the resulting sequences were assembled *de novo* using MEGAHIT (v1.2.9), retaining contigs longer than 500 bp. Assembly quality was assessed using QUAST (v5.2.0) (Gurevich et al., 2013). Predicted genes were clustered using CD-HIT (v4.8.1) to construct a non-redundant gene set. Sequencing data volumes for all samples ranged from 6.21 to 8.01 Gb, with Q20 and Q30 coverage ratios exceeding 99.51% and 97.24%, respectively, and GC content consistently above 44.41%, indicating reliable sequencing data quality.

### 2.7 Functional annotation and enrichment analysis

Species-specific annotation of the non-redundant gene set was performed using the NCBI NR database (E-value ≤ 1e−5) (Buchfink et al., 2015). Kyoto Encyclopedia of Genes and Genomes (KEGG) functional annotation was conducted using KofamKOALA (v1.3.0) software. Differences in KEGG functional abundance between groups were analyzed using the Kruskal–Wallis rank-sum test and STAMP software (v2.1.3) (Parks et al., 2014), with multiple testing corrected. KEGG metabolic pathways with corrected *p*-values < 0.05 were identified as significantly different pathways for subsequent visualization and biological functional interpretation. Since shotgun metagenomic sequencing enables direct annotation of microbial genes, this approach provides a more accurate reflection of the metabolic functional characteristics of the gut microbiota in patients with coronary heart disease compared to functional inference based on 16S rRNA.

### 2.8 Metabolomics analysis

A total of 100 μL of serum was transferred into an Eppendorf (EP) tube, followed by the addition of 400 μL of pre-cooled extraction solution (methanol: acetonitrile, 1:1 v/v) containing isotope-labeled internal standards. The mixture was vortexed for 30 s to ensure thorough mixing and was then subjected to ultrasonic extraction in an ice-water bath for 10 min to enhance metabolite recovery. Protein precipitation was performed at −40 °C for 1 h. The samples were subsequently centrifuged at 12,000 rpm for 15 min at 4 °C, and the supernatants were collected and transferred into autosampler vials. To assess the stability and reproducibility of the analytical procedure, equal aliquots of each sample supernatant were pooled to generate QC samples.

Serum metabolomic profiling was performed using a Vanquish ultra-high-performance liquid chromatography (UHPLC) system (Thermo Fisher Scientific, USA). Chromatographic separation of the analytes was achieved using a Waters ACQUITY UPLC BEH Amide column (2.1 mm × 50 mm, 1.7 μm). The mobile phase consisted of solvent A (25 mmol/L ammonium acetate and 25 mmol/L ammonia in water) and solvent B (acetonitrile). Gradient elution was conducted under the following conditions: 0–0.25 min, 95% B; 0.25–3.5 min, 95–65% B; 3.5–4.0 min, 65–40% B; 4.0–4.5 min, 40% B; 4.5–4.55 min, 40–95% B; and 4.55–6.0 min, 95% B. The column temperature was maintained at 25 °C, the autosampler at 4 °C, the flow rate at 0.5 mL/min, and the injection volume at 2 μL.

Mass spectrometric detection was performed using a high-resolution Orbitrap system controlled by Xcalibur software (v4.4, Thermo Fisher Scientific). Data were acquired in both full-scan (MS) and tandem mass spectrometry (MS/MS) modes. The optimized parameters were as follows: capillary temperature, 320 °C; sheath gas flow rate, 50 Arb; auxiliary gas flow rate, 15 Arb; full MS resolution, 60,000; MS/MS resolution, 15,000; normalized collision energy (NCE), 20/30/40; spray voltage, +3.8 kV in positive mode and −3.4 kV in negative mode; scan range, 50–1,000 Da. Raw MS data were converted to mzXML format using the ProteoWizard software (v3.0.21229, ProteoWizard Software Foundation). Subsequently, peak detection, extraction, alignment, and integration were performed using an R package based on XCMS, independently developed by Shanghai Biotree Biomedical Technology Co., Ltd. Metabolite identification was conducted by matching the acquired MS/MS spectra against the in-house BiotreeDB (v2.1) database, with an algorithm score cutoff of 0.

### 2.9 Proteomics analysis

To investigate the metabolic regulatory mechanisms in patients with CHD, proteomic profiling was performed on serum samples from both groups. Serum proteins were dissolved in an ice-water bath, followed by centrifugation at 12,000 rpm for 10 min at 4 °C, and the resulting supernatants were collected. Protein concentrations were determined using a bicinchoninic acid (BCA) protein assay kit. Subsequently, the proteins were reduced with dithiothreitol (DTT) and alkylated with iodoacetamide (IAA). A portion of each protein sample was enzymatically digested with trypsin to generate peptide fragments, which were desalted using a C18 microcolumn. From each sample, 200 ng of total peptides were separated using a nano-UPLC system (nanoElute2) and were analyzed on a timsTOF Pro2 mass spectrometer (Bruker Daltonics, Bremen, Germany) equipped with a nano-liter ion source. Data acquisition was performed in parallel mode for high-resolution peptide identification and quantification.

### 2.10 Correlation analysis between metabolomics and proteomics

To explore the associations between differential metabolites and proteins in patients with CHD and healthy controls, Spearman correlation analysis was performed. Correlation coefficient (*r*) and corresponding *p*-values were calculated for each differential metabolite–protein pair to evaluate their associations. The results were visualized as a clustered heatmap to identify potential co-regulation patterns and interaction networks between metabolites and proteins.

### 2.11 Statistical analysis

Statistical analyses were performed using SPSS version 27.0 (IBM Corp., Armonk, NY, USA). Continuous variables are presented as mean ± standard deviations. For continuous variables that met the assumptions of normality and homogeneity of variance, intergroup comparisons were conducted using independent-samples *t*-tests. Variables that did not meet these assumptions were analyzed using non-parametric tests. Categorical variables were compared using the chi-square (χ^2^) test. All statistical tests were two-sided, and a *p-*value < 0.05 was considered significant.

To assess the potential diagnostic value of significantly altered bacterial genera, metabolites, and proteins in CHD, receiver operating characteristic (ROC) curves were generated, and the area under the curve (AUC) was calculated. Sample grouping (CHD = 1, NC = 0) was used as the outcome variable, and the relative abundance of gut bacterial genera, differential metabolite levels, and differential protein expression were used as predictor variables. ROC analysis was performed using the pROC package (v1.18.0) in R software (v4.2.1), and AUC values with corresponding 95% confidence intervals (CIs) were computed. AUC values of 0.7–0.9 were interpreted as moderate diagnostic accuracy, whereas values of 0.9–1.0 indicated high diagnostic accuracy.

## 3 Results

### 3.1 Baseline characteristics, biochemical assessments, and clinical evaluations

A total of 20 participants were enrolled in this study, comprising 10 individuals in the CHD group and 10 in the NC group. The baseline characteristics, biochemical assessments, and clinical evaluations of both groups are presented in Table 1. As illustrated in Table 1, the study included both male and female participants. The sex distribution was comparable between the CHD group (five men and five women) and the NC group (four men and six women), with no significant difference observed (*p* = 1.000). No significant differences were observed between the CHD and NC groups in terms of sex distribution, heart rate, or diastolic blood pressure (*p* > 0.05). The mean age of participants in the CHD group was slightly higher than that in the NC group, with a trend toward statistical significance (*p* = 0.068). However, significant differences were observed in body mass index (BMI) and systolic blood pressure (SBP) between the two groups (*p* < 0.01).

**Table 1:** Clinical characteristics of study participants.

**Clinical and pathological indexes**	**NC (*n* = 10)**	**CHD (*n* = 10)**	***p-*values (NC vs. CHD)**
Age (years)	42.00 ± 8.10	49.80 ± 9.77	0.068
Female patients	6 (60%)	5 (50%)	1.000
Male patients	4 (40%)	5 (50%)	
BMI (18.5–23.9 Kg/m^2^)	21.09 ± 2.10	26.38 ± 1.52	*p* < 0.001
HR (60–100 bpm)	81.10 ± 10.34	82.80 ± 6.55	0.666
SBP (90–130 mmHg)	119.90 ± 8.44	133.80 ± 9.50	0.003
DBP (60–85 mmHg)	78.10 ± 5.92	84.50 ± 7.89	0.055
Coronary artery stenosis (≥ 50%)	-	59.00 ± 3.80	-
ALT (0–41 U/L)	19.32 ± 14.15	21.73 ± 11.28	0.247
AST (0–40 U/L)	18.13 ± 6.30	21.29 ± 8.43	0.280
TBIL (0–21 μmol/L)	13.34 ± 6.84	9.44 ± 3.56	0.247
DBIL (0–6.8 μmol/L)	6.19 ± 2.45	6.42 ± 1.27	0.640
IBIL (1.7–10.2 μmol/L)	4.27 ± 2.78	2.62 ± 1.74	0.130
ALB (35–52 g/L)	46.79 ± 4.07	42.66 ± 2.29	0.012
ALP (35–104 U/L)	62.61 ± 19.03	84.08 ± 14.25	0.010
γ-GT (6–42 U/L)	19.21 ± 8.18	34.01 ± 13.20	0.011
GLU (3.89–6.11 mmol/L)	4.92 ± 0.91	5.45 ± 1.12	0.260
UREA (2.86–8.21 mmol/L)	4.69 ± 0.74	5.31 ± 1.46	0.243
BUN (7–20 mg/dL)	13.12 ± 2.06	14.87 ± 4.10	0.243
CREA (45–84 μmol/L)	73.20 ± 10.58	74.86 ± 13.43	0.767
UA (142.8–339.2 μmol/L)	295.22 ± 51.75	298.79 ± 67.70	0.896
CK (0–170 U/L)	57.01 ± 17.50	85.64 ± 52.11	0.105
CK-MB (0–24 U/L)	11.73 ± 4.02	9.51 ± 3.76	0.123
LDH (109–245 U/L)	134.19 ± 21.45	150.35 ± 26.82	0.154
TG (0–2.26 mmol/L)	1.61 ± 0.97	2.89 ± 0.38	0.005
CHO (0–5.2 mmol/L)	4.54 ± 0.79	5.32 ± 0.49	0.017
HDL-C (> 0.9 mmol/L)	1.19 ± 0.25	0.99 ± 0.15	0.039
LDL-C (0–3.37 mmol/L)	2.90 ± 0.75	3.46 ± 0.60	0.081

Independent samples *t*-test was used to evaluate the differences between patients with CHD and healthy controls. Data are presented as mean ± SD or *n* (%). Significant differences between groups are indicated (*p* < 0.05). BMI, body mass index; HR, heart rate; SBP, systolic blood pressure; DBP, diastolic blood pressure; ALT, alanine aminotransferase; AST, aspartate aminotransferase; TBIL, total bilirubin; DBIL, direct bilirubin; IBIL, indirect bilirubin; ALB, albumin; ALP, alkaline phosphatase; γ-GT, γ-glutamyl transpeptidase; GLU, glucose; UREA, urea; BUN, blood urea nitrogen; CREA, creatinine; UA, uric acid; CK, creatine kinase; CK-MB, creatine kinase isoenzymes MB; LDH, lactate dehydrogenase; TG, triglyceride; CHO, cholesterol; HDL-C, high-density lipoprotein cholesterol; LDL-C, low-density lipoprotein cholesterol; SD, standard deviation; NC, healthy controls; CHD, coronary heart disease.

Regarding biochemical parameters, serum levels of ALB and HDL-C were significantly lower in the CHD group than in the NC group (*p* < 0.05). In contrast, levels of ALP, GGT, TG, and total CHO were significantly higher in the CHD group than in the NC group (*p* < 0.05). No significant differences were observed between the two groups in ALT, AST, TBIL, DBIL, IBIL, GLU, UREA, CREA, UA, BUN, CK, CK-MB, LDH, or LDL-C levels (*p* > 0.05).

### 3.2 Gut microbiota analysis

Given the significant differences in BMI and SBP between the CHD and NC groups (*p* < 0.01), as well as the potential influence of sex on gut microbiome composition, stratified analyses were conducted to evaluate their potential confounding effects. Alpha and beta diversity as well as taxonomic composition were compared across sexes (male vs female), BMI categories (BMI < 24 vs BMI ≥ 24 kg/m^2^), and SBP categories (SBP < 130 vs SBP ≥ 130 mmHg) (Jones et al., 2025), independent of CHD status. Hierarchical analysis revealed no significant differences in alpha diversity, beta diversity, or taxonomic composition at the phylum and genus levels (Supplementary Figures 1–3), suggesting that these variables are unlikely to be major confounding factors that drive differences in the microbiome between patients with CHD and healthy controls. Accordingly, subsequent analyses were focused on comparing gut microbiota diversity and composition between the CHD and NC groups.

#### 3.2.1 Alpha and beta diversity analysis

**Figure 1:**
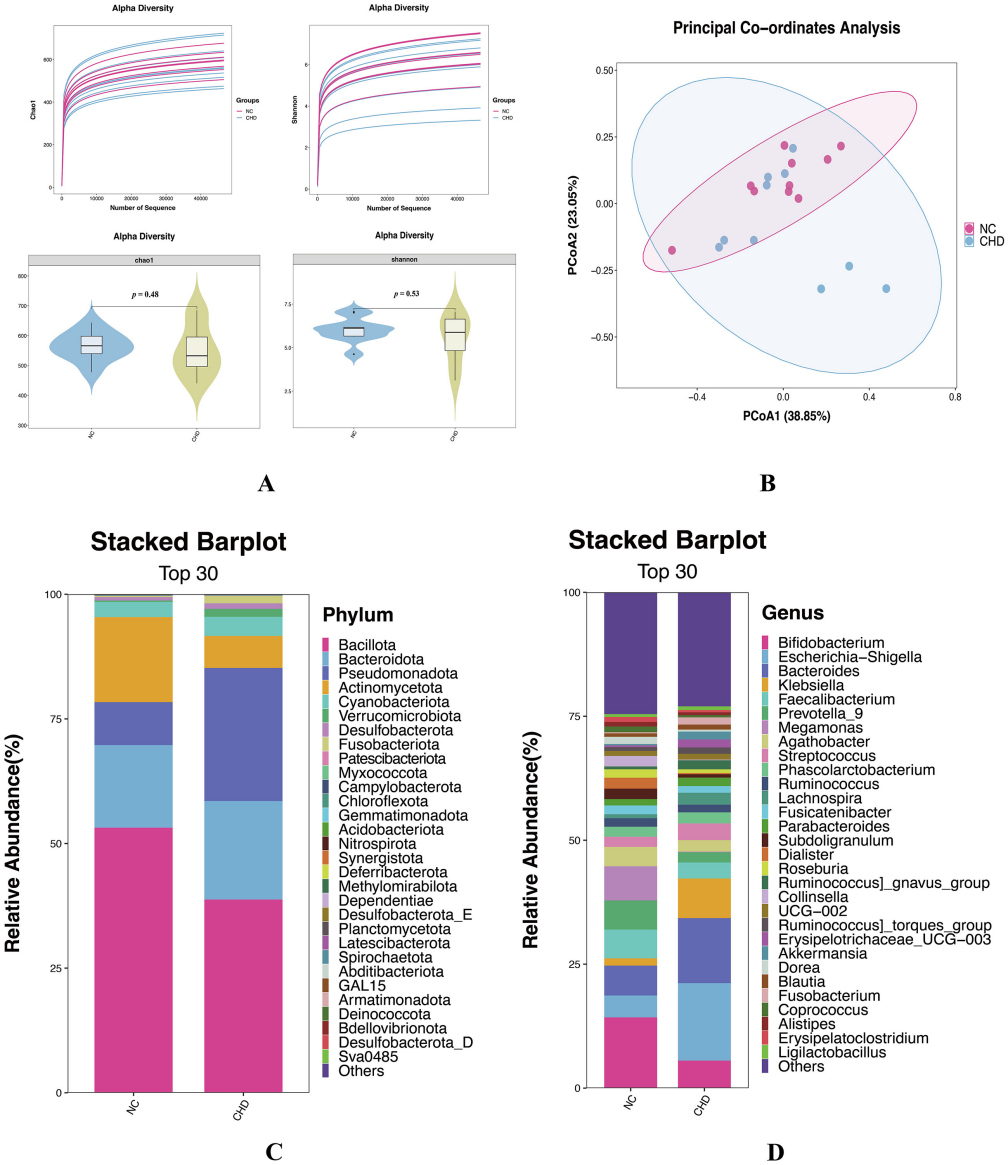
Comparison of gut microbial diversity and composition. **(A)** Alpha diversity analysis. Within-sample species diversity was assessed using the Chao 1 and Shannon indices. **(B)** Beta diversity analysis. Between-sample community differences were evaluated to determine the overall structural variation. **(C)** Phylum-level taxonomic composition. Stacked bar chart illustrating the relative abundance of the major bacterial phyla. **(D)** Genus-level taxonomic composition. Stacked bar chart depicting the relative abundance of the dominant bacterial genera. NC, healthy controls; CHD, coronary heart disease.

Alpha and beta diversity analyses were conducted to compare gut microbiota profiles between the CHD and NC groups. Alpha diversity reflects species richness and evenness within individual samples, whereas beta diversity evaluates differences in microbial community composition between samples. The rarefaction curves demonstrated sufficient sequencing depth to capture the microbial richness of all samples. Violin plots showed no significant differences in the Chao1 and Shannon indices between the CHD and NC groups (*p* > 0.05) (Figure 1A). Beta diversity, assessed using weighted UniFrac principal coordinate analysis, also revealed no significant differences in community structure between the two groups (*p* > 0.05) (Figure 1B). Non-etheless, subtle clustering patterns suggested possible ecological shifts in microbial community composition between groups.

#### 3.2.2 Species composition analysis

At the phylum level, the gut microbiota of the CHD and NC groups was dominated by Bacillota, Bacteroidota, Pseudomonadota, Actinomycetota, and Cyanobacteriota, accounting for 95.60% and 98.59% of the total taxa, respectively (Figure 1C). In the CHD group, the relative abundances of Bacteroidota (1.19-fold), Pseudomonadota (3.10-fold), and Cyanobacteriota (1.26-fold) were elevated, whereas those of Bacillota (0.73-fold) and Actinomycetota (0.38-fold) were reduced compared with those in the NC group. Notably, Pseudomonadota was significantly enriched, whereas Actinomycetota was significantly reduced in the CHD group (*p* < 0.05), indicating a shift in the overall microbial composition at the phylum level.

At the genus level, both groups were mainly composed of *Bifidobacterium, Escherichia–Shigella, Bacteroides, Klebsiella*, and *Phascolarctobacterium*, accounting for 32.11% and 45.63% of the total microbial composition in the NC and CHD groups, respectively (Figure 1D). In the CHD group, the relative abundances of *Escherichia–Shigella* (3.53-fold), *Bacteroides* (2.18-fold), and *Klebsiella* (5.23-fold) were increased, whereas those of *Bifidobacterium* (0.39-fold) and *Phascolarctobacterium* (0.56-fold) were decreased. These alterations suggest an increase in potentially pathogenic bacteria and a decrease in beneficial bacteria in patients with CHD.

#### 3.2.3 Analysis of species differences

**Figure 2:**
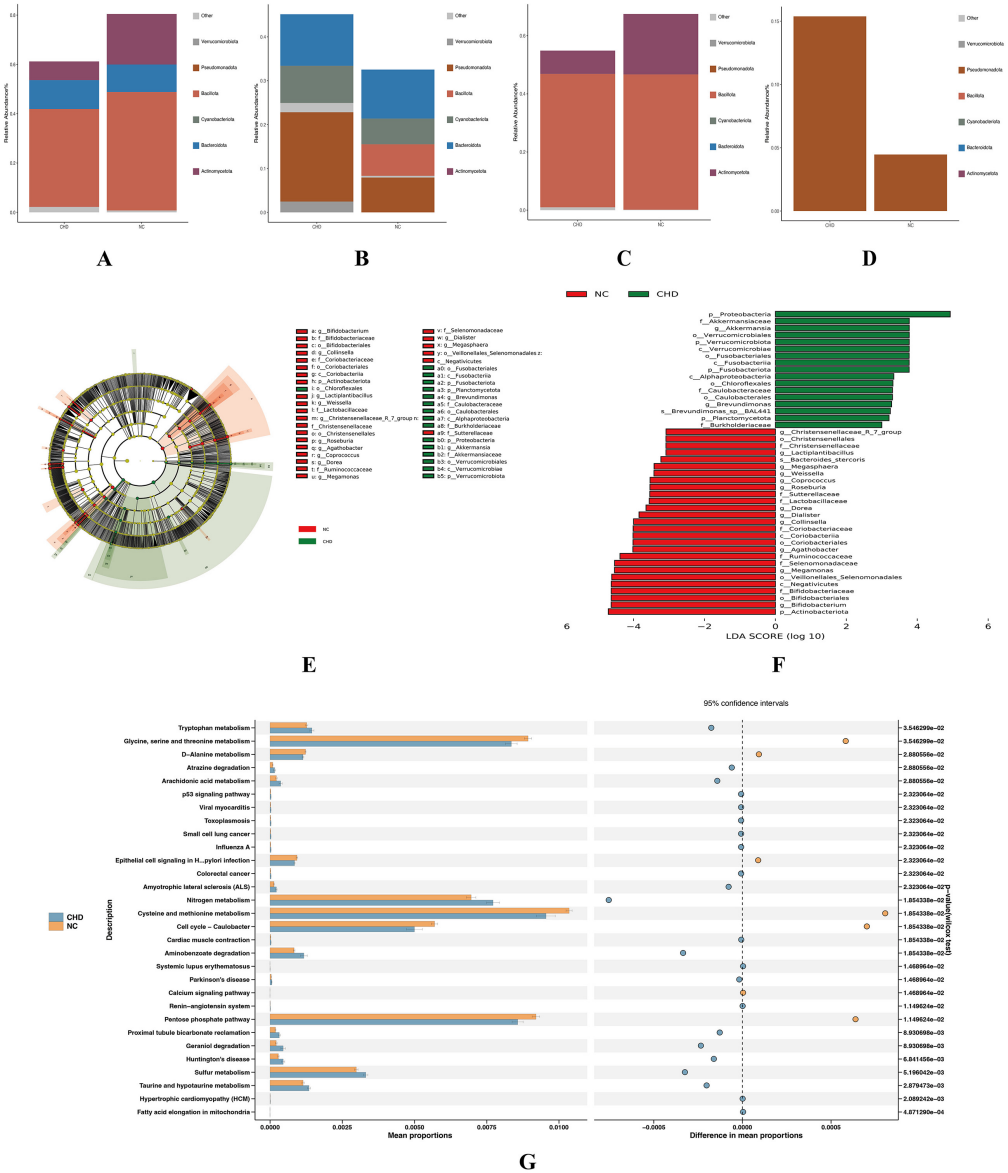
Prediction of bacterial phenotypes and LEfSe analysis. **(A–D)** Bacterial phenotype predictions. Distributions of anaerobic, gram-negative, gram-positive, and potentially pathogenic bacteria are shown. **(E)** Cladogram of LEfSe analysis. Yellow nodes represent taxa significantly enriched in the yellow-labeled group, and the other node colors follow the same pattern. **(F)** LDA score distribution. Bar length represents the LDA score, and color indicates whether taxa are more abundant in the CHD or NC group. **(G)** KEGG pathway enrichment analysis. The left bar chart shows the proportion and relative abundance of each differential function, ranked by increasing p-value. The right bubble plot illustrates functional abundance differences; dot color indicates the enriched group, and distance from the central dashed line reflects the magnitude of the difference. NC, healthy controls; CHD, coronary heart disease; KEGG, Kyoto Encyclopedia of Genes and Genomes; LDA, linear discriminant analysis.

To further characterize the phenotypic differences in gut microbiota between the CHD and NC groups, the relative abundances of anaerobic, gram-negative, gram-positive, and potentially pathogenic bacteria were predicted across samples (Figures 2A–D). In comparison with the NC group, the relative abundance of anaerobic bacteria was significantly reduced in the CHD group (*p* < 0.05), indicating an altered microbial functional profile in patients with CHD. These phenotypic alterations are consistent with the observed taxonomic shifts, including the enrichment of Pseudomonadota and the depletion of Bacillota and Actinomycetota in the CHD group.

Differential abundance analysis was performed using Linear Discriminant Analysis Effect Size (LEfSe). The cladogram illustrates the distribution of the 200 most abundant taxa across taxonomic ranks (kingdom, phylum, class, order, family, genus, and species), with node size representing relative abundance. A total of 44 taxa showed significant differences between groups via linear discriminant analysis (LDA score > 3.0, *p* < 0.05), with 27 taxa enriched in the NC group and 17 taxa enriched in the CHD group (Figures 2E, F). At the phylum level, Actinomycetota and Bacillota were significantly more abundant in the NC group, whereas Pseudomonadota, Verrucomicrobiota, and Fusobacteriota were enriched in the CHD group. These results indicate a distinct taxonomic signature associated with CHD, characterized by the enrichment of specific phyla previously linked to dysbiosis. Detailed information on all discriminating taxa is provided in Supplementary Table 1.

Based on 16S rDNA sequencing data, KEGG pathway enrichment analysis was performed using PICRUSt2. The top 30 pathways with *p* < 0.05 were identified and visualized (Figure 2G). Functional prediction revealed that the CHD group was characterized by enhanced nitrogen metabolism, aminobenzoate degradation, and sulfur metabolism, suggesting that these pathways may represent key microbiota-regulated metabolic processes involved in CHD. In contrast, the NC group showed enrichment in cysteine and methionine metabolism, cell cycle– *Caulobacter*, pentose phosphate pathway, and glycine, serine, and threonine metabolism.

#### 3.2.4 Functional forecasting analysis

**Figure 3:**
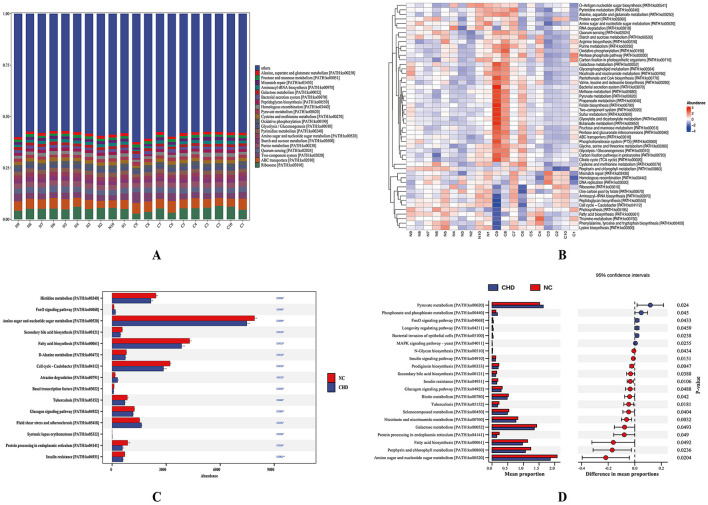
Analysis of gut microbiota KEGG functional levels between the CHD and NC groups **(A)** KEGG level 3 functional stack bar chart. The figure displays two groups of major functional categories of gut microbiota and their relative abundances. The horizontal axis represents sample names; the vertical axis represents functional proportions. **(B)** KEGG level 3 functional abundance-based clustering heatmap. This figure illustrates differences in functional distribution patterns across samples. The x-axis represents sample names; the y-axis represents functional names. **(C)** Kruskal–Wallis rank-sum test bar chart of differential pathways. Bar length indicates the abundance of KEGG functional pathways, with different colors representing the CHD group and NC group. Significantly different pathways are marked with asterisks: *p < 0.05, **p < 0.01, indicating pathways significantly enriched in either the CHD group or NC group. **(D)** STAMP analysis of differential pathway bar charts. Red bars indicate pathways significantly upregulated in the CHD group, whereas blue bars indicate pathways significantly upregulated in the NC group. The left side of the figure displays pathways with significant intergroup differences and their relative abundances in each group, whereas the right side shows the proportion of differences and 95% confidence intervals. Different colors correspond to different groups. NC, healthy controls; CHD, coronary heart disease; KEGG, Kyoto Encyclopedia of Genes and Genomes.

To investigate the functional characteristics of gut microbiota in patients with CHD, we performed shotgun metagenomic sequencing on all fecal samples, followed by KEGG functional annotation and differential functional analysis. The KEGG Level 3 functional stacked bar chart revealed overall functional similarities between the CHD and NC groups. Functional genes in both groups were primarily enriched in pathways such as ribosome, ABC transporters, two-component system, and purine metabolism (Figure 3A). Hierarchical clustering analysis of functional abundance revealed distinct distribution patterns across samples for pathways including amino sugar and nucleotide sugar metabolism, along with multiple carbohydrate and lipid metabolism-related pathways (Figure 3B). Kruskal–Wallis analysis identified 15 significantly differentially regulated pathways (Figure 3C), whereas STAMP analysis identified 21 differentially regulated pathways (Figure 3D). The metabolic pathway results filtered by functional difference analysis are detailed in Supplementary Tables 2, 3. Both analyses revealed that the forkhead box O (FoxO) signaling pathway was significantly upregulated in the CHD group, whereas pathways including fatty acid biosynthesis, secondary bile acid biosynthesis, and amino sugar and nucleotide sugar metabolism were significantly upregulated in the NC group, suggesting consistent functional differences between disease and health states.

Further analysis of the biological significance of the upregulated pathways in the CHD group revealed that the FoxO signaling pathway, mitogen-activated protein kinase (MAPK) signaling pathway, phosphonate and phosphinate metabolism, pyruvate metabolism, systemic lupus erythematosus-related pathways, longevity regulating pathway, and bacterial invasion of epithelial cells pathway were primarily involved in host energy metabolism regulation, cellular stress responses, and immune–inflammatory mechanisms. This suggests that the gut microbiota of patients with CHD may participate in disease onset and progression by coordinating metabolic and inflammatory networks. In contrast, pathways significantly upregulated in the NC group primarily relate to basal energy metabolism, lipid and carbohydrate metabolism, and insulin signaling regulation. This reflects the critical role of healthy gut microbiota in maintaining host metabolic homeostasis, energy balance, and nutrient metabolism, suggesting their contribution to preserving normal metabolic function and metabolic stability.

### 3.3 Differential metabolite analysis

**Figure 4:**
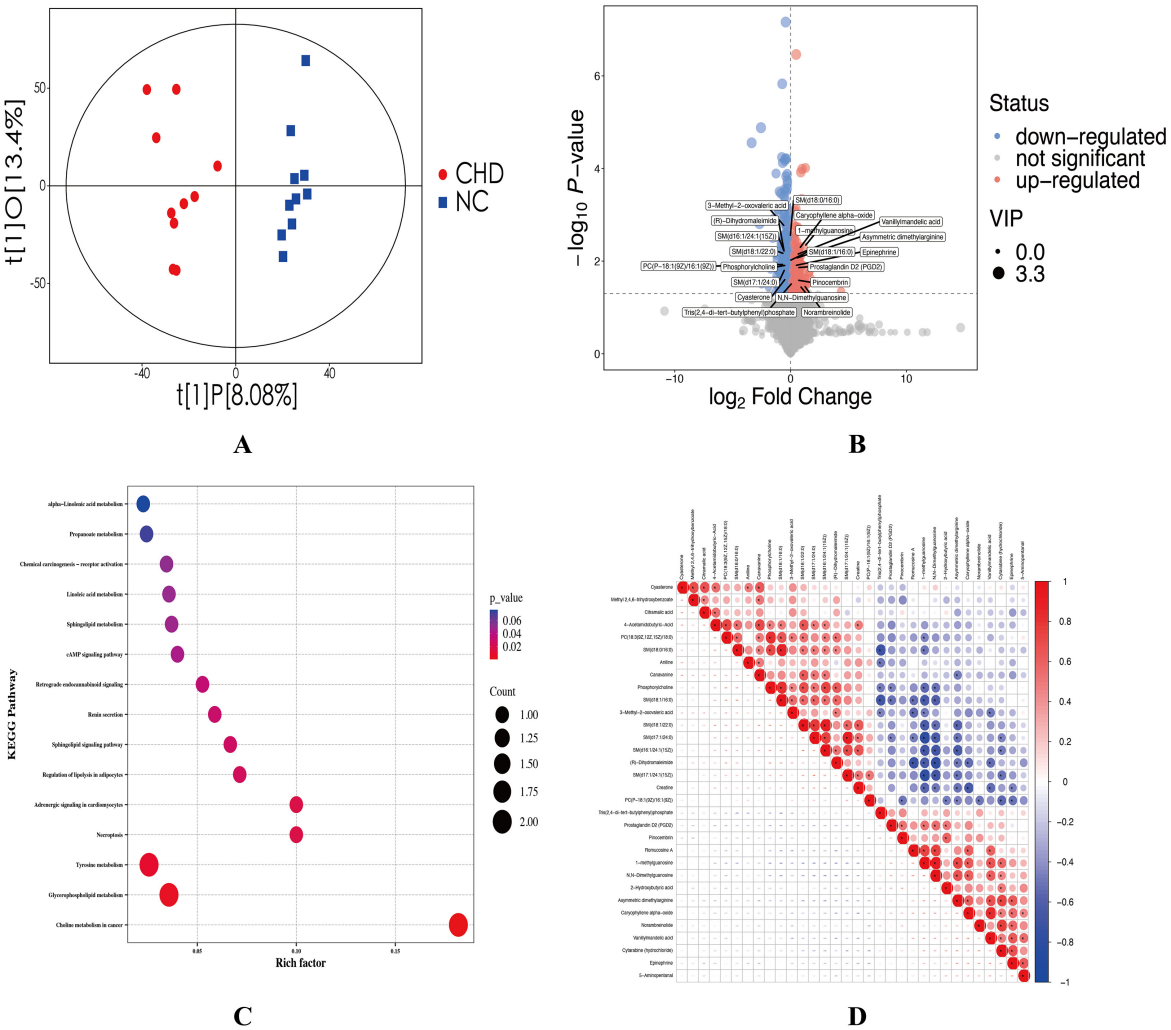
Serum metabolomics analysis of CHD and NC groups. **(A)** Score plot of the OPLS-DA model. t[1]P represents intergroup differences, and t[1]O reflects intragroup variation. The figure demonstrates differences between groups and clustering within groups. **(B)** Volcano plot of differentially expressed metabolites. A total of 32 differentially expressed metabolites were detected. The x-axis shows fold changes relative to NC, and the y-axis indicates p-values from Student's t-test. Point size reflects VIP values from OPLS-DA. Red: significantly upregulated; blue: significantly downregulated; gray: not significant. **(C)** KEGG pathway enrichment analysis of differential metabolites. Dot size indicates the number of enriched metabolites; redder color indicates lower p-value. KEGG pathway enriched in glycerophospholipid metabolism, tyrosine metabolism, and choline metabolism–related pathways. **(D)** Hierarchical clustering heatmap of differential metabolites. The heatmap shows correlation patterns among differential metabolites between the CHD and NC groups. Red indicates positive correlations and blue indicates negative correlations, with deeper colors denoting stronger correlations. Significant correlations are marked with asterisks (*). NC, healthy controls; CHD, coronary heart disease; KEGG, Kyoto Encyclopedia of Genes and Genomes; OPLS-DA, orthogonal partial least squares–discriminant analysis; VIP, variable importance in projection. ^*^p < 0.05.

Metabolites derived from gut microbiota can cross the intestinal mucosal barrier and enter the bloodstream, thereby influencing diverse physiological functions of the host. To elucidate the potential metabolic alterations associated with CHD, untargeted serum metabolomic analysis was conducted using UHPLC coupled with Orbitrap high-resolution mass spectrometry (UHPLC–OE–MS). Orthogonal Partial Least Squares-Discriminant Analysis demonstrated clear separation between the CHD and NC groups, indicating significant intergroup differences and distinct intragroup clustering in serum metabolome profiles (Figure 4A). A total of 32 differentially expressed metabolites were identified, encompassing sphingolipids, amino acids, and others (Supplementary Table 4). Among these, 14 metabolites were upregulated, whereas 18 were downregulated. In the NC group, amino acid-related metabolites were predominantly upregulated relative to those in the CHD group, whereas sphingolipid and glycerophospholipid metabolites were largely downregulated, suggesting a shift in lipid-associated metabolic profiles in CHD (Figure 4B). KEGG pathway enrichment analysis revealed that these differential metabolites were involved in 21 metabolic pathways, primarily enriched in glycerophospholipid, tyrosine, and choline metabolism in cancer (Figure 4C). Furthermore, Spearman correlation analysis was visualized via a hierarchical clustering heatmap (Figure 4D), revealing significant correlations among several differential metabolites (*p* < 0.05) and suggesting potential co-regulatory relationships in CHD-associated metabolic reprogramming.

### 3.4 Differential protein analysis

**Figure 5:**
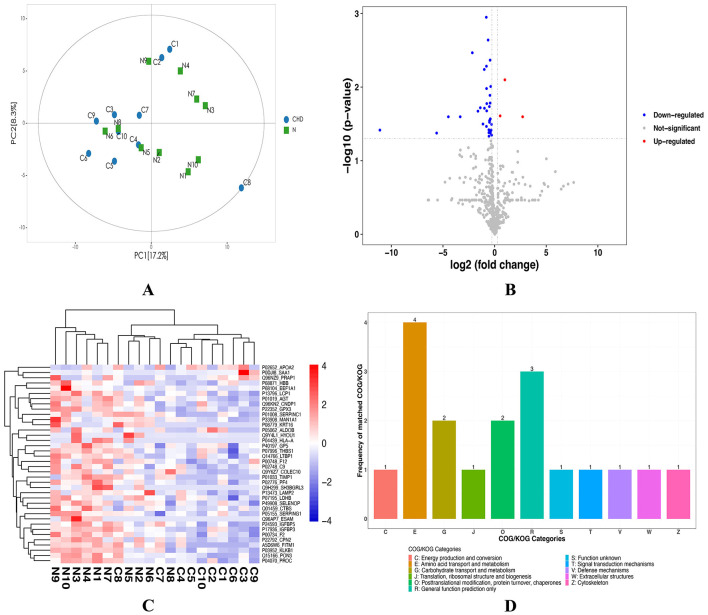
Differential protein expression analysis between CHD and NC groups. **(A)** PCA scatter plot of all samples. PC[1], and PC[2] represent the first and second principal components, respectively. The figure indicates that the two sample groups exhibit good distinguishability. **(B)** Volcano plot of differential proteins. A total of 38 DEPs were identified. Red indicates significantly upregulated proteins, blue indicates significantly downregulated proteins, and gray indicates proteins without significant changes. **(C)** Hierarchical clustering heatmap of DEPs. Red indicates high expression levels, blue indicates low expression levels, and deeper colors reflect stronger differences. Clustering demonstrates clear segregation of CHD and NC samples based on protein expression profiles. **(D)** KOG functional classification analysis. Most DEPs are associated with amino acid transport and metabolic, highlighting functional category alterations in CHD. NC, healthy controls; CHD, coronary heart disease; PCA, principal component analysis; DEPs, differentially expressed proteins; KOG, Eukaryotic Orthologous Groups; COG, Clusters of Orthologous Groups.

To further elucidate differences in metabolic mechanisms between patients with CHD and healthy controls, serum proteomic analysis was performed on both groups. Principal components analysis (PCA) indicated that all samples were distributed within the 95% CI, with a clear degree of separation between the CHD and NC groups (Figure 5A), suggesting distinct protein expression profiles. Based on the screening criteria (fold change ≥ 1.2 or ≤ 0.8; *p* < 0.05), a total of 38 DEPs were identified, comprising three upregulated proteins— apolipoprotein A-II, serum amyloid A1, and proline-rich acidic protein 1—and 35 downregulated proteins. Notably, several downregulated proteins, such as plasma kallikrein B1, antithrombin III, recombinant protein C, complement component 9, and carboxypeptidase N subunit 2, are involved in coagulation and complement pathways (Supplementary Table 5). DEPs were visualized using a volcano plot (Figure 5B), which illustrates the overall distribution of significantly DEPs between the CHD and NC groups. Hierarchical clustering heatmap analysis demonstrated that CHD and NC samples clustered distinctly according to DEP expression profiles, with a predominance of downregulated proteins in the CHD group (Figure 5C). This clustering pattern was consistent with the PCA results, thereby providing further support for the robustness of the identified proteomic differences. Eukaryotic Clusters of Orthologous Groups (KOG) functional classification showed that the DEPs were primarily enriched in amino acid transport and metabolism, as well as carbohydrate transport and metabolism (Figure 5D), suggesting alterations in metabolic and transport-related protein functions in CHD.

**Figure 6:**
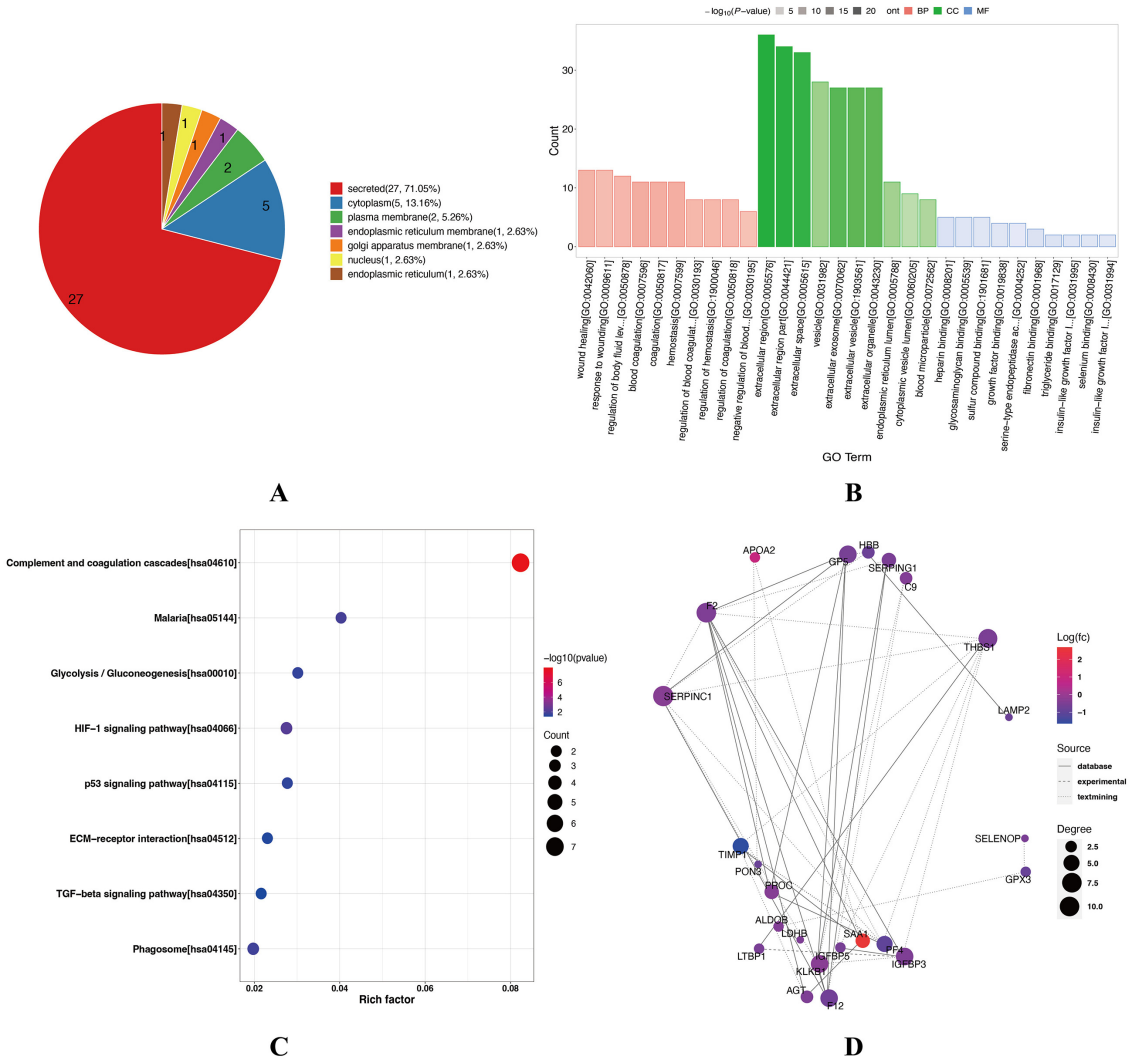
GO Functional and KEGG pathway enrichment analysis of DEPs between CHD and NC groups. **(A)** Subcellular localization analysis of DEPs. Pie chart shows the distribution of DEPs across cellular compartments, with most proteins localized to the cytoplasm. **(B)** GO functional enrichment analysis. Red indicates BP annotation information, green indicates CC annotation information, and blue indicates MF annotation information. Bar transparency represents the magnitude of the p-value: the darker the color, the smaller the p-value. **(C)** KEGG pathway enrichment analysis. Bubble chart showing pathways enriched in DEPs. The size of the circle indicates the number of DEPs in the mapped pathway; larger circles represent higher numbers. The color of the circle indicates the magnitude of the *p*-value; redder circles correspond to smaller p-values. **(D)** PPI network of DEPs. Red nodes represent upregulated proteins, whereas blue nodes represent downregulated proteins. Node size reflects connectivity, with larger nodes indicating higher interaction degrees. A total of 10 DEPs interact with F2 in the figure. NC, healthy controls; CHD, coronary heart disease; GO, Gene Ontology; KEGG, Kyoto Encyclopedia of Genes and Genomes; DEPs, differentially expressed proteins; BP, Biological Process; CC, Cellular Component; MF, Molecular Function; PPI, protein–protein interaction; F2, coagulation factor II.

Subcellular localization analysis revealed that the cytoplasm and endoplasmic reticulum membrane were the predominant sites of differential protein activity (Figure 6A). Gene Ontology (GO) enrichment analysis further characterized the functional roles of these proteins (Figure 6B). Within the biological process (BP) category, they were mainly associated with wound healing, coagulation, and hemostasis, suggesting a hypercoagulable state in CHD that may be associated with platelet activation and fibrin deposition, thereby promoting thrombosis. Cellular component (CC) analysis revealed that most proteins were localized to the extracellular region and endoplasmic reticulum lumen, whereas molecular function (MF) analysis suggested their involvement in binding to growth factors, heparin, and glycosaminoglycans. KEGG pathway enrichment analysis revealed the significant involvement of the DEPs in complement and coagulation cascades, glycolysis/gluconeogenesis, hypoxia-inducible factor-1 (HIF-1) signaling, extracellular matrix (ECM)-receptor interactions, and transforming growth factor-β (TGF-β) signaling pathway (Figure 6C). Moreover, protein–protein interaction network analysis identified coagulation factor II (F2) as a central node with strong interactions involving 10 DEPs (*p* < 0.001) (Figure 6D), suggesting its key regulatory role in the coagulation-related protein network in CHD.

### 3.5 Correlation between differentially expressed metabolites and proteins

**Figure 7:**
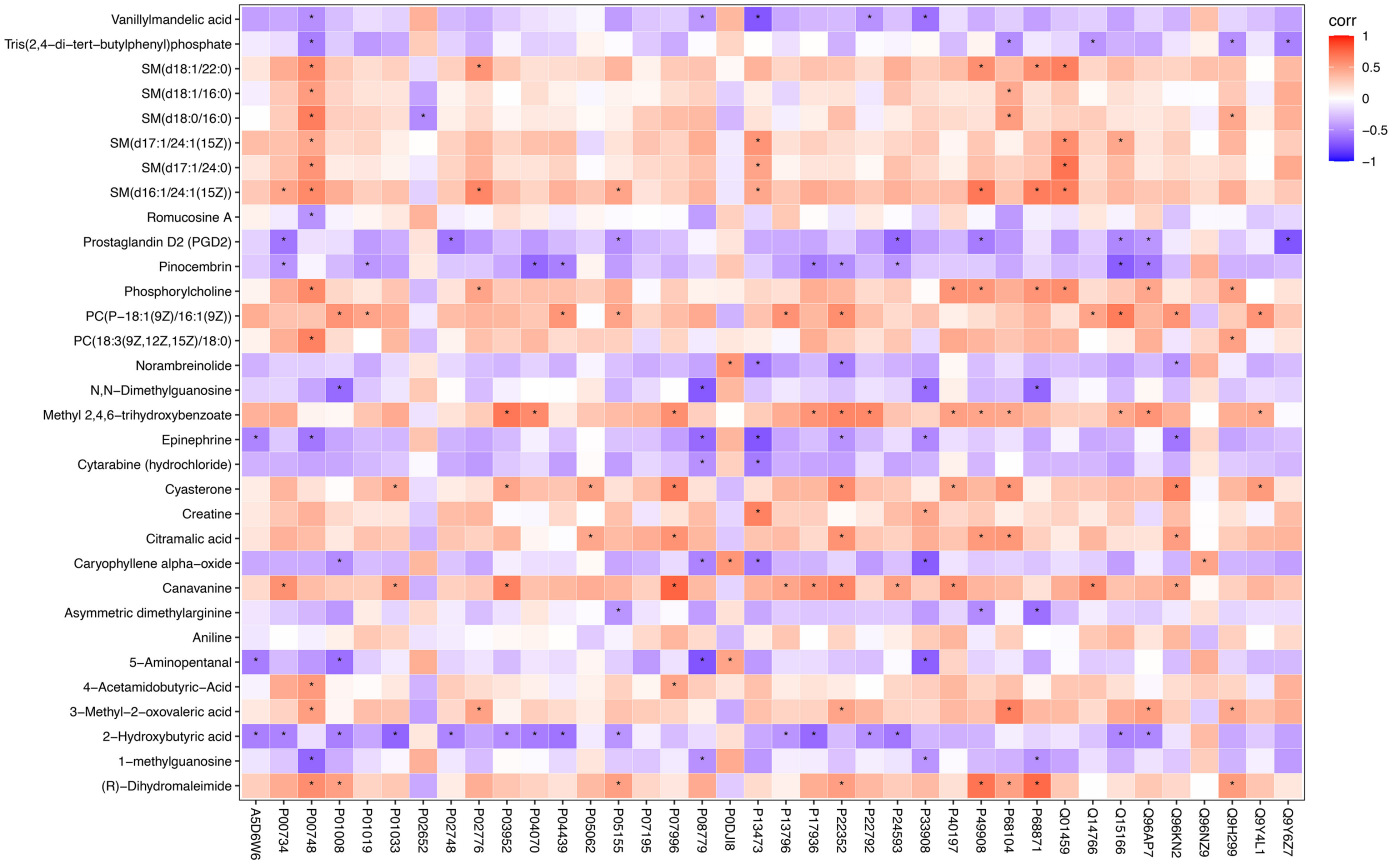
Correlation heatmap between differentially expressed metabolites and proteins. Each colored block represents the Pearson correlation coefficient between the corresponding metabolite and protein. Red indicates a positive correlation, blue indicates a negative correlation, and the color intensity reflects the strength of the correlation. Several metabolites showed significant correlations with key proteins involved in metabolic and signaling pathways, suggesting coordinated regulation at the metabolomic and proteomic levels in CHD. *p < 0.05. CHD, coronary heart disease.

To assess the relationships between differential metabolites and proteins in the NC and CHD groups, a correlation heatmap was constructed. The analysis revealed that several key metabolites exhibited significant correlation clusters with DEPs (Figure 7). Specifically, phospholipid metabolites showed positive correlations with apolipoproteins, whereas inflammatory mediators were positively associated with components of the complement system. In contrast, asymmetric dimethylarginine (ADMA) showed positive correlations with endothelial function-related proteins, and oxidative stress-related metabolites were negatively associated with antioxidant proteins (*p* < 0.05). The statistical significance of these differential metabolite–protein correlations was further evaluated, and the corresponding *p-*values are summarized in Supplementary Table 6. These findings suggest that CHD pathophysiology involves a multifactorial interplay between lipid metabolism disorders, vascular inflammation, endothelial dysfunction, oxidative stress, and energy metabolism imbalance.

### 3.6 ROC curve analysis and diagnostic value

Building on the observed alterations in gut microbiota, metabolites, and proteins (Supplementary Tables 1, 4, 5), representative differential variables were selected for ROC curve analysis to assess their potential diagnostic value for CHD. Selection criteria were as follows: gut microbiota, LDA > 3.0 and *p* < 0.05 (Yao et al., 2025); for metabolites, variable importance in projection (VIP) > 1 and fold change (FC) > 2 or FC < 0.8 with *p* < 0.05 (Ji et al., 2021; Li et al., 2023); and for proteins, |log_2_FC| > 1.0 with *p* < 0.05 (Qiu et al., 2024). Subsequently, candidate variables were refined based on their biological relevance, association with CHD, and pathway significance. ROC curves were then generated, and AUC values with their 95% CIs were calculated (Supplementary Table 7).

**Figure 8:**
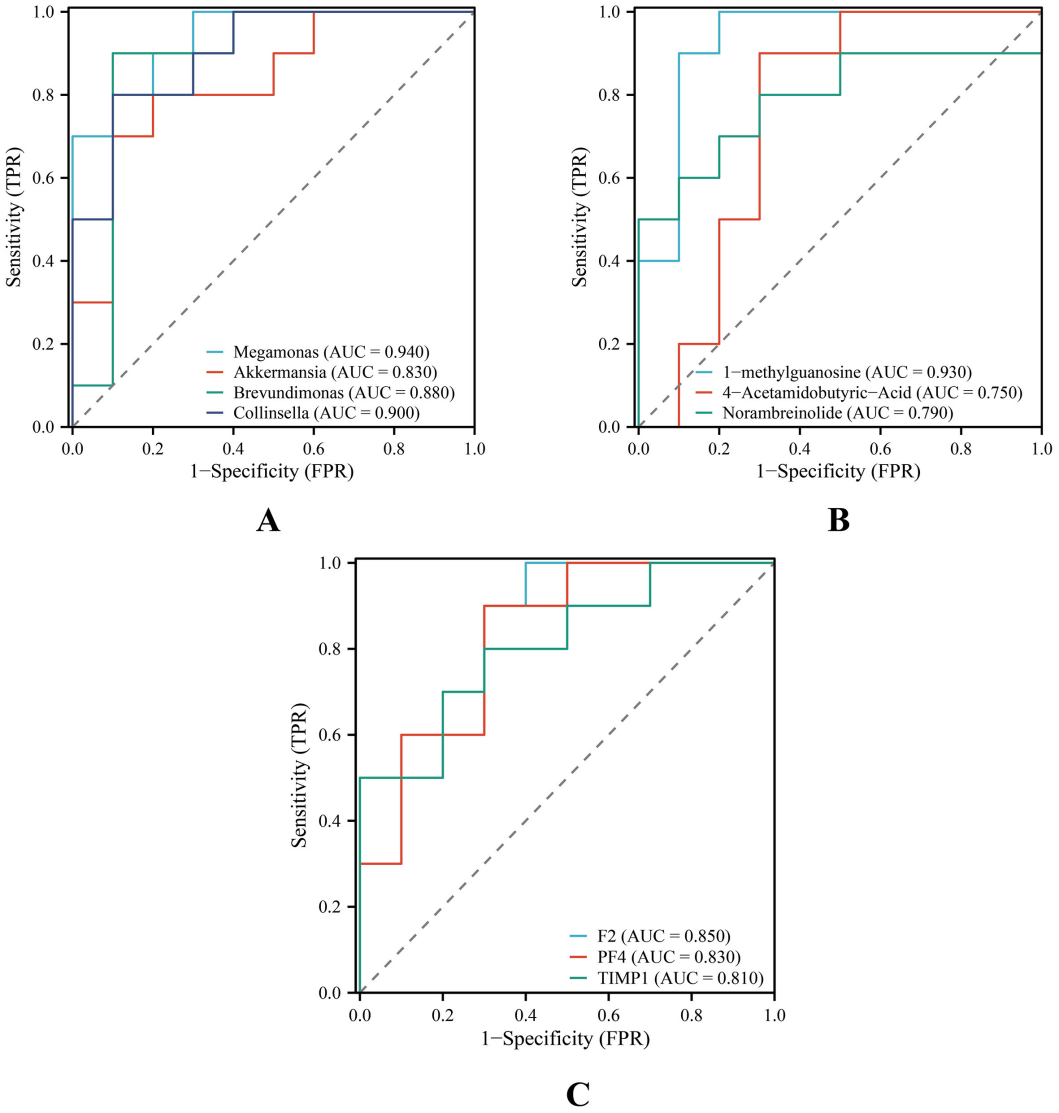
ROC curves evaluating the predictive performance of key features in each omics dataset. **(A)** Gut microbiota; **(B)** Metabolomics; **(C)** Proteomics. The ROC curve illustrates the relationship between sensitivity and specificity. The horizontal axis (X-axis) represents 1 – specificity (FPR), and the vertical axis (Y-axis) represents sensitivity (TPR). A smaller FPR and higher TPR indicate better predictive accuracy. The AUC quantifies overall diagnostic performance, with values closer to 1 indicating higher accuracy. All omics datasets demonstrated good predictive performance. F2, coagulation factor II; PF4, platelet factor 4; TIMP1, tissue inhibitor of metalloproteinases 1; FPR, false positive rate; TPR, true positive rate; AUC, area under the curve; ROC, receiver operating characteristic.

As illustrated in Figure 8, the predictive performance of differential gut microbial genera, metabolites, and proteins was evaluated using AUC values. Among the gut microbial genera, *Megamonas, Akkermansia, Brevundimonas*, and *Collinsella* showed AUC values greater than 0.800, indicating relatively strong diagnostic performance (Figure 8A). Notably, *Brevundimonas* (AUC = 0.880, 95% CI: 0.696–1.000) and *Megamonas* (AUC = 0.940, 95% CI: 0.846–1.000) showed the highest diagnostic accuracy, suggesting their potential accuracy. For metabolites, 1-methylguanosine demonstrated an AUC of 0.930 (95% CI: 0.806–1.000), indicating a strong diagnostic efficacy (Figure 8B). Among DEPs, F2 (AUC = 0.850, 95% CI: 0.678–1.000), platelet factor 4 (PF4; AUC = 0.830, 95% CI: 0.645–1.000), and tissue inhibitor of metalloproteinases 1 (TIMP1; AUC = 0.810, 95% CI: 0.616–1.000) exhibited good diagnostic performance (Figure 8C).

Collectively, these findings highlight that specific gut microbial taxa, metabolites, and proteins may serve as potential diagnostic biomarkers for CHD, supporting an integrated multi-omics approach for disease prediction and risk stratification.

## 4 Discussion

We conducted a preliminary investigation of the differential characteristics between individuals with CHD and healthy controls by integrating multi-omics datasets, including gut microbiota, untargeted metabolomics, and quantitative proteomics. The analyses revealed significant differences in gut microbiota composition, serum metabolite profiles, and protein expression between the CHD and NC groups. Moreover, correlation analyses indicated functional interactions between certain differential metabolites and proteins. These findings indicate that specific microorganisms, metabolites, and proteins may participate in metabolic regulatory processes associated with CHD, thereby providing potential biomarkers and mechanistic targets for future studies.

In the present study, 16S rRNA sequencing was employed to characterize differences in gut microbiota between patients with CHD and healthy controls. Alpha diversity analysis showed no significant differences in the Chao1 and Shannon indices between the two groups, suggesting that CHD was not associated with substantial alterations in the overall species richness or evenness of the gut microbial community. This observation aligns with previous findings that microbial dysbiosis in cardiovascular diseases is primarily driven by the enrichment of specific pathogenic taxa rather than a broad reduction in overall microbial diversity (Jie et al., 2017; Alexandrescu et al., 2024). Although beta diversity analysis revealed no significant differences, a clustering trend was observed, implying a potential ecological drift in gut microbial composition between the CHD and healthy control groups. Such structural alterations may be linked to metabolic disturbances, immune dysregulation, and inflammatory responses (Shariff et al., 2024). At the phylum level, the CHD group exhibited distinct features of gut microbial dysbiosis, characterized by increased abundances of Pseudomonadota and Bacteroidetes and reduced levels of Bacillota and Actinomycetota. Pseudomonadota, which normally constitute a minor fraction of the gut microbiota in healthy individuals, are widely recognized as indicators of microbial imbalance and are closely linked to diverse inflammatory and metabolic disorders (Shin et al., 2015; Rizzatti et al., 2017). Elevated Pseudomonadota levels facilitate endotoxin translocation into the systemic circulation, triggering proinflammatory cascades that can aggravate vascular endothelial dysfunction and accelerate atherosclerotic progression (Du et al., 2024; Wiedermann et al., 1999). Conversely, the decreased abundance of Actinomycetota may reflect a reduction in beneficial bacteria, such as Bifidobacterium, which play crucial roles in maintaining intestinal barrier integrity and producing short-chain fatty acids (SCFAs). Reduced SCFA production has been associated with low-grade inflammation and enhanced susceptibility to atherosclerosis (Karlsson et al., 2012). At the genus level, the CHD group displayed notable enrichment of opportunistic pathogens, including *Escherichia–Shigella* and *Klebsiella*. These gram-negative bacteria possess lipopolysaccharide (LPS)-rich cell walls, which can induce systemic inflammation and contribute to cardiovascular disease (Cani et al., 2008). Meanwhile, the decreased abundance of probiotic taxa, such as *Bifidobacterium* and *Faecalibacterium*, suggests a decline in host anti-inflammatory capacity and microbial homeostasis regulation, thereby fostering a gut microenvironment favorable to CHD pathogenesis (Miquel et al., 2013).

Differential species analysis revealed that the gut microbiota of individuals with CHD exhibited significant structural and functional alterations compared with those in the NC group. Both functional profiling predictions and LEfSe analysis demonstrated a substantial enrichment of gram-negative bacteria and potentially pathogenic taxa belonging to the phyla Pseudomonadota, Fusobacteriota, and Verrucomicrobiota in the CHD group, whereas the NC group was largely dominated by Bacillota and Actinomycetota. Gram-negative bacterial cell walls are rich in LPS, which can translocate into the systemic circulation through a compromised intestinal barrier, thereby activating the Toll-like receptor 4 signaling pathway, inducing systemic inflammation, and ultimately contributing to vascular endothelial injury and the progression of atherosclerosis (Gnauck et al., 2016; Ji et al., 2019). Furthermore, the CHD group exhibited dysregulated microbial metabolic functions. Enrichment of pathways related to nitrogen metabolism (including the TMA/TMAO pathway), sulfur metabolism, and aromatic compound degradation may promote oxidative stress, inflammatory responses, and the production of toxic metabolites, thereby exacerbating cardiovascular risk (Tang et al., 2013; Kondo et al., 2013; Mani et al., 2013; Nemet et al., 2020). In contrast, pathways such as the pentose phosphate pathway, cysteine and methionine metabolism, and glycine-related metabolism, which were enriched in the NC group, are known to support redox homeostasis, antioxidant defense, and cellular protection, collectively contributing to the maintenance of cardiovascular health (Fuhrer and Sauer, 2009; Paul et al., 2018; Wang et al., 2013). Overall, these findings indicate that microbial structural alterations and metabolic dysfunction in patients with CHD may contribute to cardiovascular pathophysiological processes, highlighting the potential of gut microbiota as a therapeutic target in CHD.

Building upon 16S rRNA sequencing that revealed differences in gut microbiota composition, in this study, we further employed shotgun metagenomic sequencing to analyze functional disparities in gut microbiota between patients with CHD and healthy controls. Results showed that the significantly upregulated FoxO and MAPK signaling pathway in the CHD group were closely associated with energy metabolism regulation and inflammatory response, respectively. Previous studies indicate that FoxO family transcription factors are extensively involved in oxidative stress responses, inflammation regulation, and apoptosis processes, with their abnormal activation closely linked to atherosclerosis formation and cardiovascular injury (Tsuchiya et al., 2012). The MAPK signaling pathway plays a key regulatory role in processes such as inflammatory cell recruitment, vascular endothelial dysfunction, and vascular remodeling (Totoń-Żurańska et al., 2024). Furthermore, the significantly enhanced pyruvate metabolism pathway in the CHD group suggests a potential restructuring of the gut microbiota's energy metabolism patterns. As a key intermediate in glycolysis, altered pyruvate metabolism reflects adjustments in host energy metabolism and is closely associated with metabolic reprogramming processes linked to cardiovascular disease (Fu et al., 2025). Gut microbiota regulation of this pathway may influence energy supply and metabolic signaling, thereby participating in the modulation of host cardiovascular metabolic status (Kondapalli et al., 2025). Concurrently, the enrichment of systemic lupus erythematosus-related pathways and bacterial invasion of epithelial cells in the CHD group may indicate compromised intestinal barrier function and sustained activation of chronic inflammatory responses. Studies demonstrate that impaired intestinal epithelial barriers facilitate the entry of bacterial components such as lipopolysaccharides into systemic circulation, thereby activating host inflammatory responses and inducing cardiovascular inflammation and vascular endothelial injury (Lewis and Taylor, 2020; Muttiah and Hanafiah, 2025). In contrast, fatty acid biosynthesis, secondary bile acid biosynthesis, and amino sugar and nucleotide sugar metabolism pathways were significantly upregulated in the NC group, reflecting the advantages of a healthy gut microbiota in basal energy metabolism, lipid homeostasis maintenance, and nutrient absorption. The enrichment of these functional pathways contributes to maintaining host metabolic homeostasis and provides a degree of protection against inflammatory responses and metabolic disorders (Chen et al., 2023b).

We employed untargeted metabolomic profiling using UHPLC–OE–MS to preliminarily characterize the serum metabolic alterations between CHD and healthy control groups. The results revealed a general decrease in amino acid metabolites in the CHD group, indicating disruptions in nitrogen balance and impaired amino acid utilization, which may compromise endothelial function, antioxidant capacity, and immune regulation (Kelly and Pearce, 2020). For instance, abnormal tyrosine metabolism may contribute to cardiovascular disease progression through the modulation of catecholamine synthesis and vascular tone (Eisenhofer et al., 2004), whereas reduced levels of branched-chain amino acids have been linked to impaired myocardial energy metabolism (Cheng et al., 2012). Conversely, sphingolipid and glycerophospholipid metabolite levels were significantly elevated in the CHD group. Glycerophospholipids serve as essential structural components of cellular membranes and generate degradation products, such as lysophosphatidylcholine, which have been shown to induce inflammatory responses, promote arterial stiffening, and impair endothelial integrity (Schmitz and Ruebsaamen, 2010; Su et al., 2022). KEGG pathway enrichment analysis further revealed that the differentially expressed metabolites were mainly involved in choline and glycerophospholipid metabolism. These findings suggest that, under dysregulated lipid and choline metabolism, the gut microbiota-derived conversion of dietary choline into TMA and TMAO may contribute to atherosclerotic progression (Wang et al., 2011). Moreover, Pearson correlation analysis demonstrated significant covariation among several differential metabolites, implying that these metabolites may be interconnected within shared metabolic networks or are regulated by a common biochemical pathway. Collectively, these results provide a metabolic framework for identifying candidate biomarker combinations and elucidating the metabolic underpinnings of CHD pathogenesis.

Quantitative proteomic analysis identified 38 significant DEPs, delineating serum protein expression profiles specific to patients with CHD. GO functional enrichment analysis revealed that these DEPs are predominantly involved in BPs related to wound healing, hemostasis, and coagulation, aligning with the hypercoagulable state commonly observed in CHD (Borissoff et al., 2010). This finding underscores the contribution of chronic inflammation, platelet activation, and fibrin deposition to atherosclerotic plaque formation (Alkarithi et al., 2021). MF analysis has demonstrated enrichment in growth factors, heparin, and glycosaminoglycan binding, suggesting potential involvement in vascular remodeling, smooth muscle cell proliferation, and endothelial adhesion processes (Hinoki et al., 2010; Gopal, 2020). CC analysis further indicated that the DEPs were primarily localized to the extracellular region and endoplasmic reticulum lumen, suggesting that these proteins may influence systemic inflammation and metabolic homeostasis through secretory or membrane-associated pathways. Moreover, endoplasmic reticulum stress has been closely linked to CHD pathogenesis and can trigger macrophage pyroptosis, thereby exacerbating atherosclerotic progression (Zhang et al., 2023; Ren et al., 2021). These proteomic findings highlight key biological pathways underlying CHD, offering mechanistic insights into the interplay between coagulation, inflammation, and vascular dysfunction. KEGG pathway enrichment analysis further revealed that the DEPs were significantly enriched in complement and coagulation cascades, glycolysis/gluconeogenesis, HIF-1 signaling, ECM–receptor interaction, and TGF-β signaling pathways. Among these, the complement and coagulation cascades reflect the cross-activation of inflammatory and coagulation systems within atherosclerotic lesions (Zhao et al., 2016), whereas the HIF-1 and TGF-β signaling pathways are involved in myocardial hypoxia responses and vascular remodeling, playing key roles in coronary artery stenosis and ischemic preconditioning (Semenza, 2014; Hu et al., 2018). Protein–protein interaction network analysis revealed that F2 exhibits close associations with multiple DEPs. F2 serves as a central coagulation factor in thrombosis and modulates inflammatory responses and endothelial dysfunction through protease-activated receptors, thereby acting as a key driver of atherosclerosis and representing a potential biomarker (Rajput et al., 2025).

By integrating metabolomic and proteomic data, we uncovered potential interactions between metabolic reprogramming and protein expression regulation during CHD development, predominantly characterized by dysregulated lipid metabolism, inflammation activation, endothelial dysfunction, and oxidative stress. Notably, phospholipid metabolites were positively correlated with apolipoproteins, suggesting that lipid metabolic disturbances play a critical role in CHD pathogenesis. As structural components of lipoproteins, aberrant phospholipid metabolism can impair HDL-mediated reverse cholesterol transport, accelerate atherosclerotic plaque formation (Ozerova et al., 2007), and exacerbate cardiovascular injury by activating immune-mediated pro-inflammatory pathways (Tabas et al., 2015). Additionally, inflammation-related metabolites showed a positive correlation with complement system proteins, indicating a reciprocal relationship between metabolic dysregulation and immune activation. Previous studies have shown that the complement system is activated during the early stages of atherosclerosis and contributes to plaque rupture and thrombosis formation (Maffia et al., 2024). Metabolic intermediates may further modulate immune signaling, amplifying complement activation and intensifying local inflammation and endothelial injury. Furthermore, ADMA was negatively correlated with endothelium-related proteins, suggesting that it may mediate endothelial dysfunction by inhibiting NO synthesis, impairing vascular relaxation, and promoting endothelial cell apoptosis (Böger, 2005; Papageorgiou et al., 2023). Finally, multiple oxidative stress-related metabolites were negatively correlated with antioxidant enzymes, reflecting compensatory depletion of antioxidant defenses in patients with CHD. This finding aligns with the established role of redox imbalance in plaque instability and vascular remodeling (Fraley et al., 2009; Trpkovic et al., 2015).

In this study, ROC analysis was conducted as a validation step based on differential screening results. As the bacterial genera, metabolites, and proteins included in the ROC analysis were derived from previously identified differential features (Supplementary Tables 1, 4, 5, the findings are biologically coherent and mutually reinforcing. The results demonstrated that multiple indicators possessed strong discriminative power. Notably, *Brevundimonas* achieved 90% sensitivity, specificity, and accuracy at the optimal cutoff value, highlighting its potential as a promising microbial biomarker for early CHD detection. *Brevundimonas* species are classified as opportunistic pathogens (Ryan and Pembroke, 2018) and have been implicated in bacteremia and infective endocarditis in clinical case reports (Soto et al., 2022). Limited experimental evidence suggests that *Brevundimonas* may influence intestinal barrier integrity and inflammatory responses (Li et al., 2025). Similarly, an elevated abundance of *Megamonas* has been reported in patients with coronary atherosclerosis, which is associated with SCFA metabolism (Dong et al., 2023). In populations at risk for cardiovascular disease, increased abundance of *Megamonas* has also been linked to metabolic abnormalities, such as dyslipidemia and obesity, as well as alterations in SCFA production (Fadhillah et al., 2025). These findings are partly consistent with our results, supporting the hypothesis that *Megamonas* may contribute to CHD-related metabolic homeostasis regulation through gut microbiota–metabolite interactions. At the metabolite level, 1-methylguanosine exhibited strong diagnostic performance. Elevated levels of this protein are likely associated with oxidative stress and nucleotide metabolism disturbances, consistent with previous studies identifying its predictive value for cardiovascular events (Bøgh et al., 2022). This observation aligns with the amino acid and phospholipid metabolic imbalances identified in our analysis. At the protein level, F2, PF4, and TIMP1 demonstrated strong discriminative ability, consistent with prior reports indicating that patients with CHD are characterized by coagulation pathway activation, enhanced platelet degranulation, and ECM remodeling (Baidildinova et al., 2021). Overall, these multi-omics studies provide mutually reinforcing evidence that CHD development may result from the synergistic interplay among gut microbiota dysbiosis, metabolic pathway perturbations, and activation of the coagulation–inflammation axis.

However, this study has some limitations. First, the relative sample size in each group was 10, which limited statistical power and may have led to an underestimation of certain omics-based differences or failure to reach statistical significance. In particular, the number of significantly altered metabolites and proteins was limited, which may reflect both sample size constraints and high inter-individual variability in metabolic and proteomic profiles, as well as the use of stringent statistical thresholds following multiple comparison corrections. Second, as a single-center retrospective cohort study, the generalizability and representativeness of these findings require validation in independent, external populations. The stability and applicability of key biomarkers across diverse demographic and clinical backgrounds remain to be determined. In addition, detailed information on lifestyle factors, including diet, physical activity, smoking status, and alcohol consumption, was not systematically collected. Although participants underwent standardized health examinations, residual confounding by lifestyle factors cannot be excluded. Finally, although the multi-omics approach provided insights into potential pathogenic pathways and functional molecules associated with CHD, these findings remain correlative. Functional validation through mechanistic studies using animal models or *in vitro* experiments is necessary to substantiate causal relationships. Therefore, future studies should employ larger, multicenter cohorts with longitudinal or interventional designs to further elucidate the molecular mechanisms underlying CHD and validate the diagnostic and therapeutic potential of the identified biomarkers.

In conclusion, we conducted a preliminary multi-omics analysis of individuals with CHD by integrating 16S rRNA sequencing, shotgun metagenomic sequencing, untargeted metabolomics, and quantitative proteomics. The results revealed an increased abundance of Pseudomonadota and several opportunistic pathogens in the gut microbiota of patients with CHD, accompanied by reduced SCFA-producing bacteria, indicating the presence of gut microbial dysbiosis. Shotgun metagenomic functional profiling revealed significant changes in the metabolic potential of microbes. Metabolomic profiling demonstrated a general downregulation of amino acid-related metabolites and a significant upregulation of phospholipid and sphingolipid metabolites, suggesting dysregulation of energy metabolism and membrane lipid homeostasis. Proteomic analysis revealed altered expression of multiple proteins associated with complement activation and coagulation, indicating a state of chronic low-grade inflammation and hypercoagulability in CHD. Correlation analysis identified potential functional links between differentially expressed metabolites and proteins, whereas ROC curve analysis highlighted *Brevundimonas, Megamonas*, 1-methylguanosine, and several coagulation- and inflammation-related proteins as candidate biomarkers with high discriminative performance. Overall, this study provides preliminary evidence of the microbial, metabolic, and proteomic features associated with CHD, offering novel insights into its early diagnosis and risk stratification.

## Data Availability

The gut microbiota 16S rRNA gene sequencing and metagenomic sequencing data presented in this study have been deposited in the NCBI repository, with corresponding accession numbers PRJNA1421073 and PRJNA1423428.
