# Supplementary material for: Integrated multi-omics analysis reveals gut microbiota and metabolic characteristics in coronary heart disease
Source: Front Microbiol. 2026 Mar 10;17:1743914. doi: 10.3389/fmicb.2026.1743914 (PMC13008861; doi:10.3389/fmicb.2026.1743914)
Supplement: Supplementary file 1 [file Data_Sheet_1.pdf]

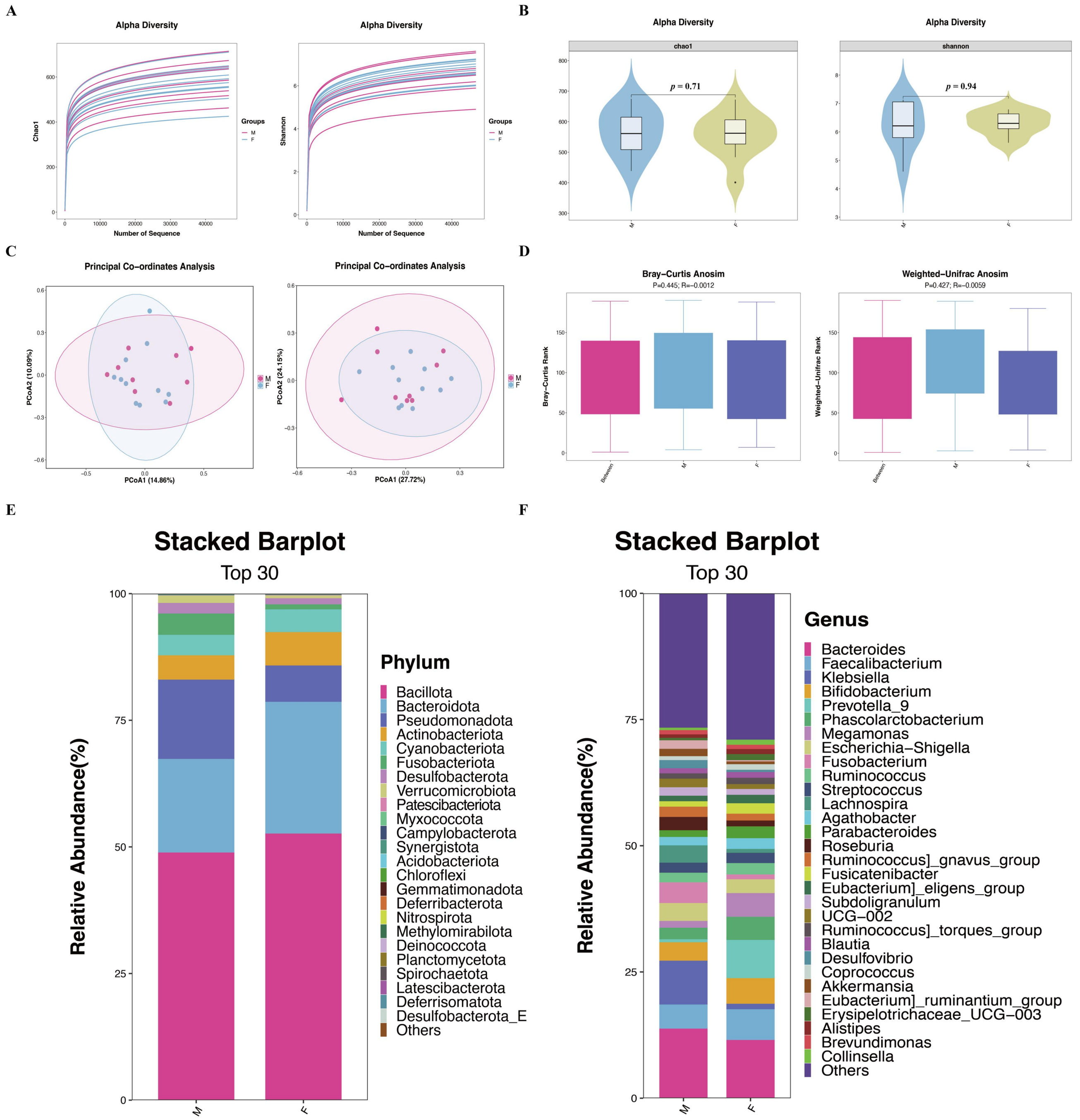

Supplementary Figure 1. Comparison of gut microbiota diversity and composition between male (M) and female (F) participants. **(A-B)** Alpha diversity analysis. Within-sample species diversity was assessed using the Chao 1 and Shannon indices. **(C-D)** Beta diversity analysis. Between-sample community differences were compared using Bray-Curtis and weighted UniFrac algorithms to determine overall structural changes. **(E-F)** Phylum and genus-level taxonomic composition. Stacked bar charts illustrating the relative abundance of major bacterial phyla and genera. Abbreviations: M, male; F, female. *$p < 0.05$.

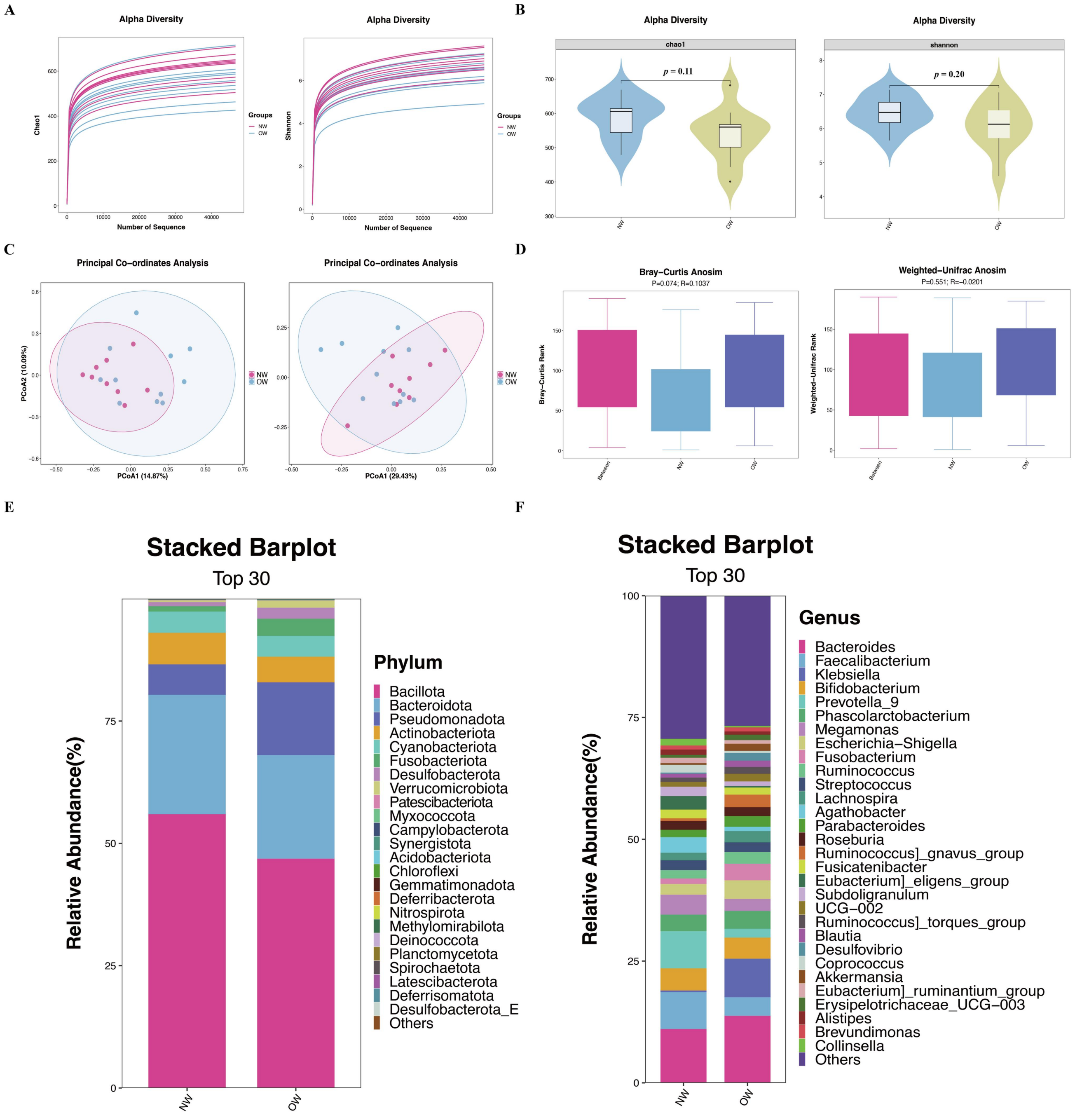

Supplementary Figure 2. Comparison of gut microbiota diversity and composition between normal weight (NW) and overweight (OW) participants. **(A-B)** Alpha diversity analysis. Within-sample species diversity was assessed using the Chao 1 and Shannon indices. **(C-D)** Beta diversity analysis. Between-sample community differences were compared using Bray-Curtis and weighted UniFrac algorithms to determine overall structural changes. **(E-F)** Phylum and genus-level taxonomic composition. Stacked bar charts illustrating the relative abundance of major bacterial phyla and genera. Abbreviations: NW, normal weight; OW, overweight. *$p < 0.05$.

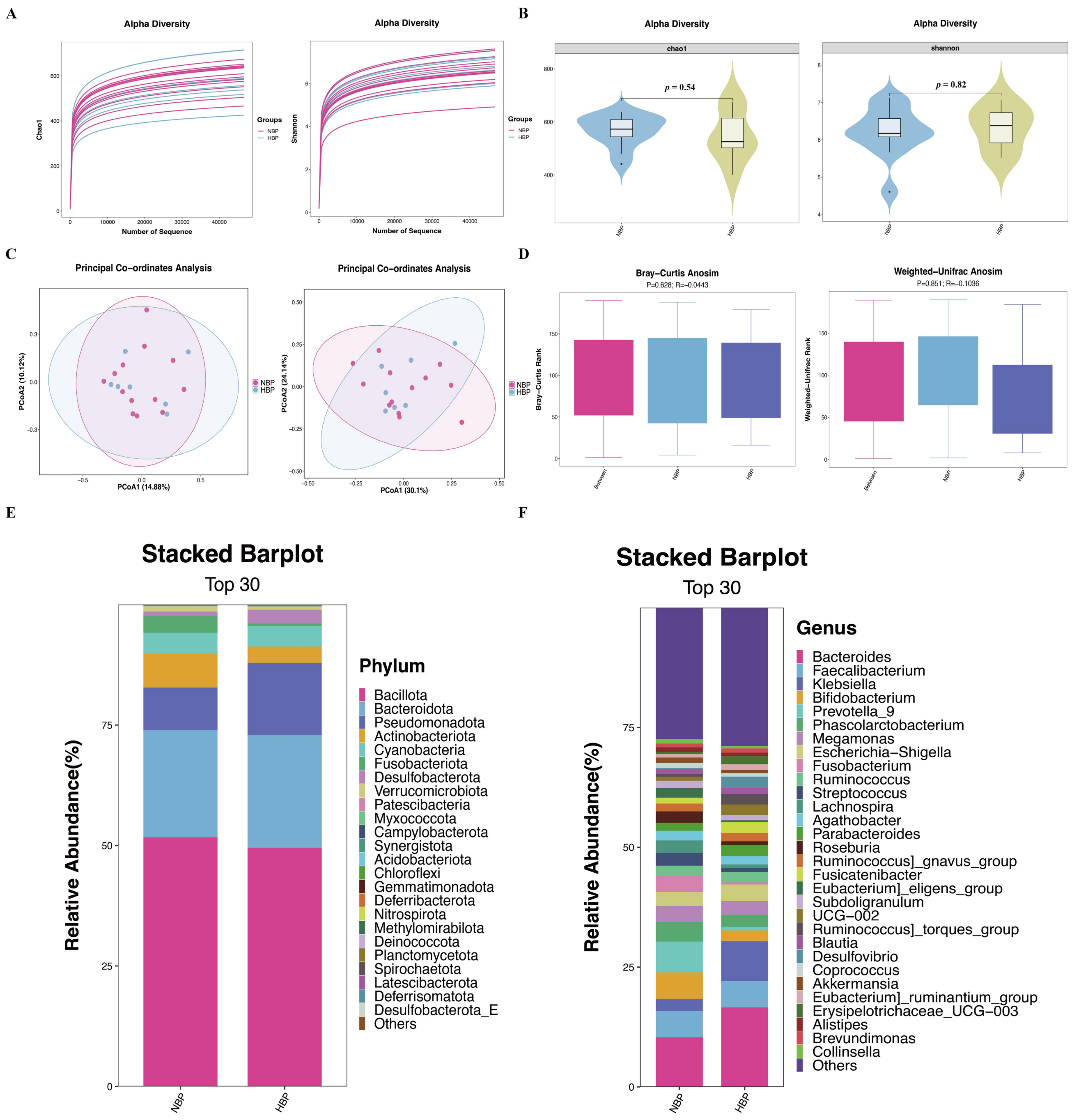

Supplementary Figure 3. Comparison of gut microbiota diversity and composition between participants with normal blood pressure (NBP) and those with high blood pressure (HBP). **(A-B)** Alpha diversity analysis. Within-sample species diversity was assessed using the Chao 1 and Shannon indices. **(C-D)** Beta diversity analysis. Between-sample community differences were compared using Bray-Curtis and weighted UniFrac algorithms to determine overall structural changes. **(E-F)** Phylum and genus-level taxonomic composition. Stacked bar charts illustrating the relative abundance of major bacterial phyla and genera. Abbreviations: NBP, normal blood pressure; HBP, high blood pressure. *$p < 0.05$.
